# Retinal Electrophysiology Is a Viable Preclinical Biomarker for Drug Penetrance into the Central Nervous System

**DOI:** 10.1155/2016/5801826

**Published:** 2016-04-27

**Authors:** Jason Charng, Zheng He, Algis J. Vingrys, Rebecca L. Fish, Rachel Gurrell, Bang V. Bui, Christine T. Nguyen

**Affiliations:** ^1^Department of Optometry & Vision Sciences, University of Melbourne, Parkville, VIC 3010, Australia; ^2^Pfizer Neusentis, Cambridge CB21 6GS, UK

## Abstract

*Objective*. To examine whether retinal electrophysiology is a useful surrogate marker of drug penetrance into the central nervous system (CNS).* Materials and Methods*. Brain and retinal electrophysiology were assessed with full-field visually evoked potentials and electroretinograms in conscious and anaesthetised rats following systemic or local administrations of centrally penetrant (muscimol) or nonpenetrant (isoguvacine) compounds.* Results*. Local injections into the eye/brain bypassed the blood neural barriers and produced changes in retinal/brain responses for both drugs. In conscious animals, systemic administration of muscimol resulted in retinal and brain biopotential changes, whereas systemic delivery of isoguvacine did not. General anaesthesia confounded these outcomes.* Conclusions*. Retinal electrophysiology, when recorded in conscious animals, shows promise as a viable biomarker of drug penetration into the CNS. In contrast, when conducted under anaesthetised conditions confounds can be induced in both cortical and retinal electrophysiological recordings.

## 1. Introduction

The annual cost of treating central nervous system (CNS) diseases in the United States has grown rapidly from US$250 billion in 2007 [1] to more than US$750 billion in 2014 [2]. This growth is expected to further accelerate as longer life expectancy [3] increases the incidence of age-related neurodegenerative disorders [4, 5]. One strategy to reduce the cost involved in CNS drug development is to create preclinical biomarkers that help to triage centrally penetrant compounds in the animal testing phase [6]. Ideally these surrogate measures should be translatable into the clinic to confirm efficacy of compounds in future human trials.

The brain shares many similarities with the retina, the sensory lining of the eye. Both organs are derived from the same tissue during foetal development [7] and show similar blood neural barriers (blood retinal barrier, BRB, and blood-brain barrier, BBB) comprised of comparable tight junctions [8–10]. Blood vessels in the retina and brain are similarly affected by ageing and by many diseases, including diabetes and high blood pressure [11–14]. Indeed, changes to the retinal vasculature are associated with increased risk of stroke in patients with hypertension [14]. Furthermore, the major neurotransmitters (i.e., glutamate, *γ*-aminobutyric acid, and glycine) and their associated receptors involved in neural signal transmission are found in both the brain and the retina [15–17]. This raises the possibility that the retina, which is a more accessible organ than the cortex, may provide a viable brain biomarker [18, 19] for testing drug penetrance through the BBB.

One way to confirm central penetrance is to measure electrophysiological responses from locations of interest in the brain. One example is the electroencephalogram, which has been utilised to test activity of candidate compounds in preclinical and clinical settings [20–22]. Other well-defined electrophysiological measures may also be useful in this regard, including evoked potentials. One evoked potential that may be particularly useful is the electroretinogram (ERG), which is the combined light evoked electrical activity from a range of retinal neurons. Decomposition of the ERG into its constituent parts provides an index of the function of specific retinal cell classes [23, 24]. Moreover, specific receptor classes produce well-defined and repeatable changes to various components of the ERG response. For these reasons, centrally penetrant CNS drugs that target a receptor type found in both the eye and the brain should produce measurable and specific changes in the ERG. Interestingly, a recent report by Lavoie et al. [25] suggests the possibility of using the ERG as a biomarker of central dopamine and serotonin levels. In addition to the ERG, the visually evoked potential (VEP) may also be useful for this purpose. The VEP is a summation of light evoked excitatory and inhibitory postsynaptic potentials in the visual cortex [26], arising from cortical neurons whose apical dendrites are perpendicularly orientated relative to the scalp [27]. It provides a measure of cortical responsiveness to light originating from the retina serially transmitted via the optic nerve, optic tract, thalamus (lateral geniculate nucleus, LGN), and optic radiation to cortical area V1 [28].

Unlike clinical settings, preclinical ERG and VEP recordings are conventionally performed under anaesthesia, which ensures stable placement of electrodes in an animal model. However, anaesthesia can alter neuronal responses [29–32], by acting directly or indirectly on receptor systems [33, 34]. Thus anaesthesia may present a significant confound to drug penetrance testing. To overcome the need for anaesthesia we have developed a platform for wireless ERG and VEP recordings in conscious rats [32]. This recording platform will be used to determine if systemic administration of a centrally penetrant drug, targeting receptors known to exist in both the eye and the brain, produces measurable changes to the ERG and VEP. If so, this would provide evidence that the centrally penetrant drug has crossed both the blood-brain and blood retinal barriers.

To test the above hypothesis, isoguvacine and muscimol are employed. Both compounds are predominantly GABAa (*γ*-aminobutyric acid type a) receptor agonists with one key difference being that isoguvacine does not cross the blood neural barriers readily, whereas muscimol easily traverses these barriers [35, 36]. In rats, GABAa receptors are found in both the retina [37, 38] and the visual cortex [39]. Thus systemic (i.e., intramuscular, IM) application of muscimol should result in ERG and VEP changes similar to those seen following direct injection of the drug (i.e., intravitreal, IV, or intracerebroventricular, ICV), whereas little change should be seen following intramuscular injection of isoguvacine. Furthermore, if the findings in the eye (ERG) mirror those found in the brain (VEP) following systemic administration of drugs then the BRB and BBB exhibit similar penetrance characteristics. Such a finding would support the notion of using the retina as a biomarker for drug penetrance into the CNS. To determine how a commonly used laboratory anaesthesia might influence drug testing, the same dosing experiments were repeated in rats anaesthetised with ketamine : xylazine. To complement the functional data, pharmacokinetic (PK) analysis was conducted to determine whether the presence of anaesthesia could alter drug penetrance through the blood neural barriers.

## 2. Material and Methods

### 2.1. Ethics Statement

All experiments were conducted in accordance with the Australian Code of Practice for the Care and Use of Animals for Scientific Purposes set out by the National Health and Medical Research Council. Animal ethics approval was obtained from the Animal Ethics Committee of the Faculty of Science, The University of Melbourne.

### 2.2. Animal Preparations

Long-Evans rats (male, 3 to 4 months old) were raised in a controlled environment, with ambient temperature maintained at 21°C and a 12-hour light/dark cycle (on at 8 am, maximum illuminance < 50 lux at the top of the cage). Animals were randomised into those used for recordings under anaesthetised or conscious states. For ERG/VEP recordings in conscious animals, two cohorts of rats (*n* = 7 for each group) were used, one for muscimol and the other for isoguvacine injections. All injection routes (i.e., intramuscular, IM; intravitreal, IV; intracerebroventricular, ICV) were performed in the same rats with at least 3 days of recovery between each injection protocol. The order in which drug injections were performed was determined via a pseudorandom block design. To ensure that there was no cumulative effect of isoguvacine or muscimol, ERG was measured at −2.3 log cd·s·m^−2^ prior to each injection and the same was done for VEPs (measured at 1.52 log cd·s·m^−2^). Unpaired *t*-tests of the ERG or VEP measured before drug application on the day and sham baseline returned no significant differences (ERG *p* = 0.31 to 0.72, VEP *p* = 0.11 to 0.66), suggesting that the 3-day interval between each injection route allowed adequate recovery.

For recordings under anaesthetised conditions (intramuscular ketamine : xylazine, 60 : 5 mg/kg, Troy Laboratories Pty Ltd., Smithfield, NSW, Australia), each injection route was performed in a separate cohort (*n* = 5 per group) as lengthy and/or repeated anaesthesia could lead to complication such as perioperative respiratory distress [40].

Prior to electrophysiological recordings rats were dark-adapted overnight. Animals were prepared for recordings with the aid of a dim red light (LED 22 lux @ 10 cm, *λ*_max_ = 650 nm). Mydriasis was induced with one drop of tropicamide (0.5%, Alcon Laboratories, Frenchs Forest, NSW, Australia) and topical anaesthesia with one drop of proxymetacaine (0.5%, Alcon Laboratories) prior to recordings.

### 2.3. ERG and VEP Recordings in Conscious Rats

For recordings in conscious animals, rats were implanted with telemetry transmitters (F50-EEE, Data Sciences International, St. Paul, MN, USA). The surgical techniques and assessment of implant stability have previously been reported [32]. Briefly, the F50-EEE transmitter (bandwidth: 1–100 Hz) has three recording channels (for more details please see Charng et al. [32] for signal amplification): two channels were used to record ERG from each eye while the third channel was fixed over one randomly chosen visual cortex via a stainless screw (diameter 0.7 mm, length 3 mm, Micro Fasteners Pty Ltd., Thomastown, VIC, Australia) for VEP recording. All active electrodes were referenced to an inactive stainless steel screw electrode secured on the skull midline, 5 mm rostral to bregma. This electrode arrangement allowed simultaneous assessment of retinal and cortical responses to light in the same animal. As 90% of the ganglion cell axons synapsing at the lateral geniculate nucleus decussate to the contralateral hemisphere in rats [41], it is possible to measure VEPs dominated by one eye without occluding the other eye [42]. Rats recovered for a week following surgery before overnight dark-adaptation and recording.

### 2.4. ERG and VEP Recordings in Anaesthetised Rats

Rats were anaesthetised with intramuscular injection of ketamine : xylazine and body temperature was maintained at 37.5 ± 0.5°C by placing the animal on a platform with circulating heated water (Techne Inc. Temperature Junior TE-8J, Burlington, NJ, USA). Anaesthetised ERGs were recorded by placing custom-made chlorided silver electrodes on the eyes (active electrode on the corneal apex and inactive electrode ring around the sclera behind the limbus) and a stainless steel needle inserted into the tail which served as the ground electrode. See He et al. [43] for details.

Anaesthetised VEPs were measured from stainless steel screw electrodes implanted at the same stereotaxic coordinates as the conscious preparation (active electrode 7 mm caudal to bregma, 3 mm lateral to midline, and inactive electrode 5 mm rostral to bregma on midline). The ground was a stainless steel needle electrode (F-E2-30 Grass Telefactor, West Warwick, RI, USA) inserted into the tail. The procedure for implanting the skull electrodes in the anaesthetised preparation replicated that used for conscious implantation of electrodes. See Tsai et al. [42] for further details regarding electrode implantation.

### 2.5. Light Stimulus

The stimulus system consists of light-emitting diodes embedded into a Ganzfeld integrating sphere (Photometric Solutions International, Huntingdale, VIC, Australia), which delivered even illumination to the retina. ERG signals were collected from low to high luminous energies (−5.6 to 1.52 log cd·s·m^−2^), with progressively fewer signals averaged and longer interstimulus intervals at higher luminous energies. A twin-flash paradigm [44], employing an interstimulus interval of 500 ms, is shorter than the refractory time of the rod pathway at 1.52 log cd·s·m^−2^, allowing isolation of the cone response [24]. Finally, a VEP signal was returned from the average of 20 flashes at 1.52 log cd·s·m^−2^ with an interstimulus interval of 5 seconds. For conscious recordings, rats were placed in a custom-made clear container which allows the eyes to face the opening of the Ganzfeld sphere [32].

### 2.6. Drug Delivery

The same concentration of isoguvacine (Tocris Biosciences, Ellisville, MO, USA) or muscimol (Bachem, Bubendorf, Switzerland) was injected in both anaesthetised and conscious rats. The following doses were used: isoguvacine IM (30 mg/kg), IV (12 mM at the vitreous), and ICV (3 mM at the lateral ventricle) and muscimol IM (6 mg/kg), IV (0.2 mM at the vitreous), and ICV (0.02 nM at the lateral ventricle), assuming 45 *μ*L vitreous volume [45] and 90 *μ*L cerebrospinal fluid volume [46].

The control signals and amplitudes reported in this study were determined from the combination of recordings made following vehicle sham injections (Milli-Q water, Merck Millipore, Billerica, MA, USA) into the muscle (IM), vitreous (IV), or brain (ICV) of rats. Note that the conscious and anaesthetized control cohorts formed two different groups and thus were not combined.

#### 2.6.1. Systemic Dosing

All systemic drugs were delivered via intramuscular injections. Isoguvacine or muscimol was administered at 0.5 mL/kg. A high intramuscular dosage was chosen to increase the likelihood of the drug reaching the retina and/or brain thus producing a robust signature.

#### 2.6.2. Intravitreal Dosing

Drugs were introduced into the vitreous to bypass the blood-retina barrier. As previously described [47], a 30 G needle was connected via a length of polyethylene tubing (inner diameter 0.38 mm, Portex Limited, Kent, UK) to a Hamilton syringe (SGE® Analytical Sciences Pty Ltd., Ringwood, VIC, Australia). The needle was inserted into the vitreal chamber 2 mm behind the limbus at a 45° angle to a depth of 2.5 mm. For recordings in anaesthetised rats, injection was undertaken following placement of the inactive ring electrode. For recordings in conscious rats, intravitreal injections were performed under topical anaesthesia (proxymetacaine 0.5%, Alcon Laboratories) with one experimenter gently retracting the eyelids and the second performing the injection.

#### 2.6.3. Intracerebroventricular Dosing

Drugs were introduced directly into the lateral ventricle to bypass the blood-brain barrier. This was achieved by injecting the drug using a needle to a depth of 3.5 mm through a small hole drilled 2 mm caudal to bregma and 2 mm lateral to midline. For recordings in anaesthetised rats the skin and periosteum overlying the skull were removed, a hole was drilled over the lateral ventricle coordinates, and the drug was delivered via a 30 G needle connected to polyethylene tubing and a Hamilton syringe. For recordings in conscious rats a cannulation port was implanted on the skull (C313GFL4/SP, Plastics One, Roanoke, VA, USA) during telemetry transmitter implantation. This infusion system allows direct injection of isoguvacine or muscimol into the lateral ventricle in conscious rats, with one experimenter stabilising the animal while the other injecting the compound.

### 2.7. Pharmacokinetics Study

Pharmacokinetic analysis was performed to investigate whether anaesthesia altered the CNS penetrance of the systemically delivered compounds. For isoguvacine and muscimol age- and sex-matched rats (*n* = 5 for each group) underwent intramuscular dosing under conscious or anaesthetised (ketamine : xylazine) conditions. Tissue was harvested 90 minutes after drug administration to match the end point of electrophysiology measurements. Brain, retina, and vitreous tissues were collected immediately after stunning and decapitation.

Isoguvacine and muscimol concentrations in each tissue were analysed with a liquid chromatography-tandem mass spectrometer (API5000, Sciex, Framingham, MA, USA) and compared against precalibrated measures for these compounds [48, 49].

### 2.8. Analysis of Electroretinogram Signals

The ERG procedure has been described in detail by Weymouth and Vingrys [24]. Below is a summary of the analytical approaches.

#### 2.8.1. Photoreceptor Response

The leading edge of the scotopic a-wave can be described by a delayed Gaussian [22] as formulated by Hood and Birch [50] and based on the model of Lamb and Pugh Jr. [51]:

(1) P3i,t=RmP3·1−exp⁡−i·S·t−td2,t>td.

Equation (1) gives the photoreceptor response (P3, *μ*V) for a given luminous energy (*i*, log cd·s·m^−2^) as a function of time after flash onset (*t*, ms) by its saturated amplitude (*Rm*_P3_, mV) and sensitivity (*S*, log m^2^·cd^−1^·s^−3^). The delay (*t*_*d*_, s) term largely reflects delays in the recording equipment [52, 53]. Given that different hardware is used for recordings from conscious and anaesthetised rats, *t*_*d*_ was fixed to the average delay for the specific recording hardware (7.40 ms for conscious recordings and 4.75 ms for anaesthetised recordings) determined from control eyes [43]. The model was optimised to the leading edge of the raw ERG a-wave amplitude by floating *Rm*_P3_ and *S* to minimise the sum-of-square error using the Solver module (Microsoft*™*, Redmond, WA, USA) across an ensemble response to the two highest energies (1.20, 1.52 log cd·s·m^−2^).

#### 2.8.2. Rod Bipolar Cell Response

The putative rod bipolar cell response (P2) was isolated by subtracting the cone bipolar response (from twin-flash paradigm) and the photoreceptor model (see (1)) from the raw ERG at the highest luminous energy (1.52 log cd·s·m^−2^). Luminous energies below −1.38 log cd·s·m^−2^ have previously been shown to contain minimal cone input [24, 54]; hence the waveforms returned at these luminous energies can be considered to be rod dominant.

A saturating hyperbolic function [55] was modelled across these rod-dominant responses:

(2) $$\begin{array}{c} V\left(\;i\;\right)=V_{\max}\frac{i}{i+k}, \end{array}$$

where the P2 amplitude (*V*, *μ*V) as a function of luminous energy (*i*, log cd·s·m^−2^) is given by its saturated amplitude (*V*_max⁡_, *μ*V) and semisaturation constant (*k*, log cd·s·m^−2^). The Solver module was used to minimise the sum-of-square error term by floating *V*_max⁡_ and *k*.

#### 2.8.3. Cone Response

The cone b-wave returned from the twin-flash paradigm was analysed by taking its peak amplitude (mV) and implicit time (ms).

### 2.9. Analysis of Visually Evoked Potentials

P1, N1, and P2 were extracted from each VEP waveform. These three landmarks are defined as the first three distinct features of the VEP waveform consistent with the literature [28, 42, 56].

### 2.10. Statistical Comparisons

All group data were summarised as average ± SEM. The data for all sham injections (IM, IV, and ICV routes in conscious animals) were pooled into a single control group, to maximise sensitivity to detect drug effects. The same was done for all sham injections in the anaesthetised cohort. All data were expressed as a percentage change relative to the conscious or anaesthetised sham average (±SEM, %). Unpaired *t*-tests were performed between drug injections and the control cohort for ERG/VEP parameters, with an alpha value of 0.05 for statistical significance.

## 3. Results

### 3.1. ERG Changes following Drugs Injections in Conscious Rats

**Figure 1:**
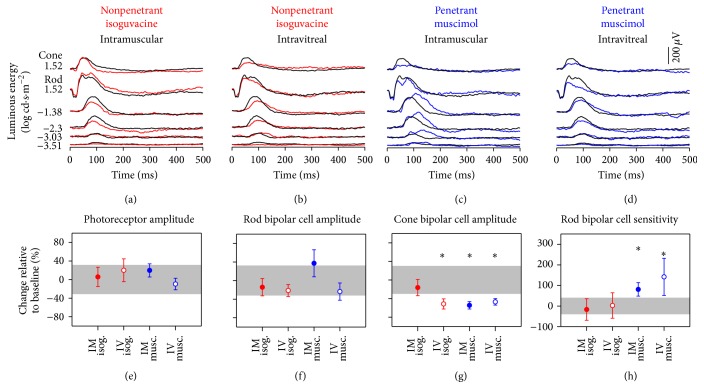
Retinal electrophysiology following conscious isoguvacine/muscimol dosing. ERG responses in conscious animals ((a)–(d), *n* = 7 for each drug group) at baseline (black lines, average of vehicle injections in both conscious groups) compared to (a) intramuscular and (b) intravitreal injection of isoguvacine (red) and (c) intramuscular and (d) intravitreal injection of muscimol (blue). (e)–(h) summarise ERG parameters (average ± SEM) following isoguvacine or muscimol injections via the different delivery routes (see method for details). Grey areas indicate 95% CI of the particular ERG parameter in all conscious baseline recordings. ^*∗*^ *p* < 0.05.

Figure 1 shows that in conscious rats the ERG b-wave can be reliably detected at −3.51 log cd·s·m^−2^. At the brightest light level the ERG shows the expected profile, with a corneal negative a-wave followed by the rod b-wave. The b-wave shows two distinctive peaks: one at 45 ms and a second between 60 and 90 ms after flash onset. The rat cone waveform (top most waveform) contains a single broad b-wave, which peaks at approximately 65 ms.

Intramuscular isoguvacine injection in conscious rats produced little effect on the ERG (Figure 1(a)), as confirmed in the summary of key parameters (Figures 1(e)–1(h), filled red circles within 95% confidence interval of sham treatment shaded). Intravitreal injection of isoguvacine resulted in a slight reduction of the rod b-wave at moderate luminous energies (Figure 1(b), −3.51 to −1.38 log cd·s·m^−2^). At the highest luminous energy, the early peak of the b-wave appeared unchanged, whereas the slower peak was smaller. These effects did not reach statistical significance (Figures 1(e) and 1(f), *p* = 0.39 to 0.96), with the exception of the cone b-wave, which was significantly reduced following IV injection of isoguvacine (Figure 1(g), −51 ± 11%, *p* < 0.05).

Figure 1(c) shows that IM injection of muscimol produced a marked b-wave double peak at low and moderate light levels (−3.03 to −1.38 log cd·s·m^−2^). At high luminous energies the first peak appeared smaller and faster, whereas the second b-wave peak was larger than in controls. This accounts for the significant increase in rod P2 sensitivity (Figure 1(h), 81.0 ± 32.6%, *p* < 0.05), with no change in rod P2 amplitude (Figure 1(f), 37 ± 29%, *p* = 0.23). There was a marked decrease in cone amplitude following IM injection of muscimol (Figure 1(g), −55 ± 8%, *p* < 0.05).

Changes to the ERG seen following IV injection of muscimol in conscious rats (Figure 1(d)) were similar to those observed after IV isoguvacine injection (Figure 1(b)). There was no change to photoreceptor (Figure 1(e), −10 ± 12%, *p* = 0.67) and rod bipolar (Figure 1(f), −24 ± 18%, *p* = 0.37) amplitudes. Cone bipolar cell amplitude was smaller (Figure 1(g), −47 ± 7%, *p* < 0.05). There was a significant increase in rod bipolar cell sensitivity following IV muscimol (Figure 1(h), 142 ± 90%, *p* < 0.05).

### 3.2. ERG Changes following Drug Injections in Anaesthetised Rats

**Figure 2:**
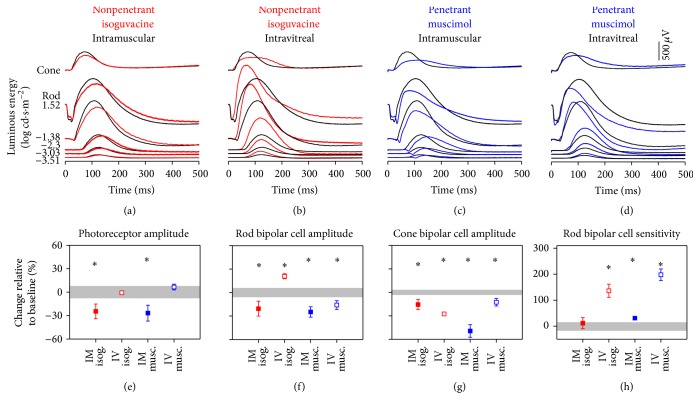
Retinal electrophysiology following anaesthetised isoguvacine/muscimol dosing. ERG responses in anaesthetised animals ((a)–(d), *n* = 5 for each drug group) at baseline (black lines, average anaesthetised groups) compared to (a) intramuscular and (b) intravitreal isoguvacine (red) and (c) intramuscular and (d) intravitreal muscimol (blue). (e)–(h) summarise ERG parameters (average ± SEM) following isoguvacine or muscimol injections via different routes (see method for details). Grey areas indicate 95% CI of the particular ERG parameter in all anaesthetised baseline recordings. Note the different scale bar in (h). ^*∗*^ *p* < 0.05.

Administration of isoguvacine and muscimol in anaesthetised rats (Figure 2) produced the following ERG changes that were different from those seen in conscious rats (Figure 1). Firstly, there was a decrease in rod photoreceptor amplitude following IM injection of isoguvacine (Figure 2(e), −25 ± 10%, *p* < 0.05) and muscimol (−27 ± 10%, *p* < 0.05). Secondly, rod bipolar cell amplitude was smaller following IM injection of isoguvacine (Figure 2(f), −21 ± 10%, *p* < 0.05). Lastly, IV injection of isoguvacine in anaesthetised rats (Figure 2(b)) produced faster and larger rod bipolar responses (Figure 2(f), 21 ± 3%, *p* < 0.05) as well as increased sensitivity (Figure 2(h), 136 ± 25%, *p* < 0.05).

Figure 2(c) shows that intramuscular injection of centrally penetrant muscimol produced a reduction in the photoreceptoral a-wave, a slowing of the b-wave at low to moderate light levels (<−2.3 log cd·s·m^−2^), and a faster but smaller b-wave at higher light levels (>−1.38 log cd·s·m^−2^). The cone ERG was reduced and prolonged. This pattern of change is similar to that seen with IV injection of the same drug (Figure 2(d)). Specifically, intravitreal injection of muscimol in anaesthetised rats did not affect photoreceptor output but there was a decrease in rod b-wave amplitude (Figure 2(f), −16 ± 6%, *p* < 0.05) with increased sensitivity (Figure 2(h), 198 ± 23%, *p* < 0.05). Cone b-wave amplitudes were smaller when drugs were injected in anaesthetised rats (Figure 2(g), IM isoguvacine −16 ± 7%, IV isoguvacine −28 ± 1%, IM muscimol −49 ± 8%, and IV muscimol −13 ± 5%, all *p* < 0.05).

### 3.3. VEP Response following Drug Administration in Conscious and Anaesthetised Rats

#### 3.3.1. VEP Changes following GABA Agonist Injection in Conscious Rats

**Figure 3:**
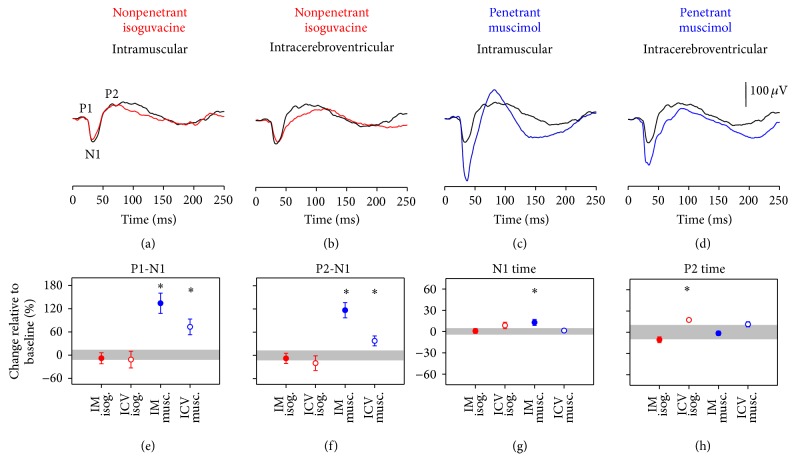
Cortical electrophysiology following conscious isoguvacine/muscimol injections. VEP responses in animals ((a)–(d), *n* = 7 for each drug group) at conscious baseline (black lines, average of vehicle injections in conscious groups) compared to (a) intramuscular and (b) intravitreal injection of isoguvacine (red) and (c) intramuscular and (d) intravitreal injection of muscimol (blue). (e)–(h) summarise VEP parameters (average ± SEM) following isoguvacine or muscimol injections via different routes (see method for details). Grey areas indicate 95% CI of the particular VEP parameter in all conscious baseline recordings. P1, P2, and N1 are marked in (a), with P2 taken as the first positive inflection following N1. ^*∗*^ *p* < 0.05.

Intramuscular injection of isoguvacine in conscious rats had little effect on the VEP waveform (Figure 3(a)) or its parameters as shown in Figures 3(e)–3(h) (*p* = 0.14 to 0.74). Direct ICV injection of isoguvacine (Figure 3(b)) significantly delayed the P2 component of the VEP (Figure 3(h), 17 ± 1%, *p* < 0.05). P2-N1 amplitude was not significantly affected (Figure 3(f), −20 ± 19%, *p* = 0.21).

Intramuscular injection of centrally penetrant muscimol in conscious rats (Figure 3(c)) substantially changed the VEP. Both P1-N1 (Figure 3(e), 134 ± 26%, *p* < 0.05) and P2-N1 amplitudes (Figure 3(f),  117 ± 20%, *p* < 0.05) were increased and N1 was significantly delayed (Figure 3(g),  13 ± 4%, *p* < 0.05) compared to sham. ICV injection of muscimol affected the VEP waveform in a way similar to IM muscimol (Figure 3(d)). Specifically, P1-N1 amplitude (Figure 3(e), 134 ± 26%, *p* < 0.05) and P2-N1 (Figure 3(f), 117 ± 20%, *p* < 0.05) were both larger; however N1 timing was not significantly affected (Figure 3(g), 1 ± 2%, *p* = 0.61).

#### 3.3.2. VEP Changes following GABA Agonist Injection in Anaesthetised Rats

**Figure 4:**
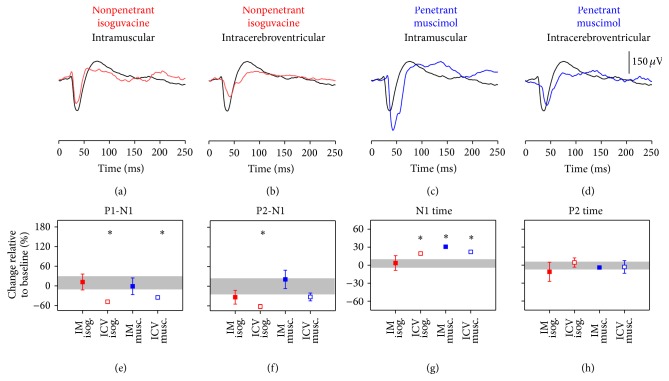
Cortical electrophysiology following anaesthetised isoguvacine/muscimol dosing. VEP responses in animals ((a)–(d), *n* = 5 for each drug group) at anaesthetised baseline (black lines, average of anaesthetised groups) compared to (a) intramuscular and (b) intravitreal isoguvacine (red) and (c) intramuscular and (d) intravitreal muscimol (blue). (e)–(h) summarise VEP parameters (average ± SEM) following isoguvacine or muscimol injections via different routes (see method for details). Grey areas indicate 95% CI of the particular VEP parameter in all anaesthetised baseline recordings. ^*∗*^ *p* < 0.05.

Intramuscular injection of isoguvacine had little effect on the VEP (Figure 4(a)), whereas ICV injection (Figure 4(b)) resulted in smaller amplitudes (Figures 4(e)–4(h), P1-N1 −49 ± 4%, P2-N1 −62 ± 6%, both *p* < 0.05) and delayed N1 implicit time (Figure 4(g), 19 ± 2%, *p* < 0.05). IM and ICV administration of muscimol significantly delayed N1 implicit times (IM, 31 ± 2%, ICV, 22 ± 3%, both *p* < 0.05). Other VEP parameters were not affected.

### 3.4. Pharmacokinetics (PK) Analysis

**Figure 5:**
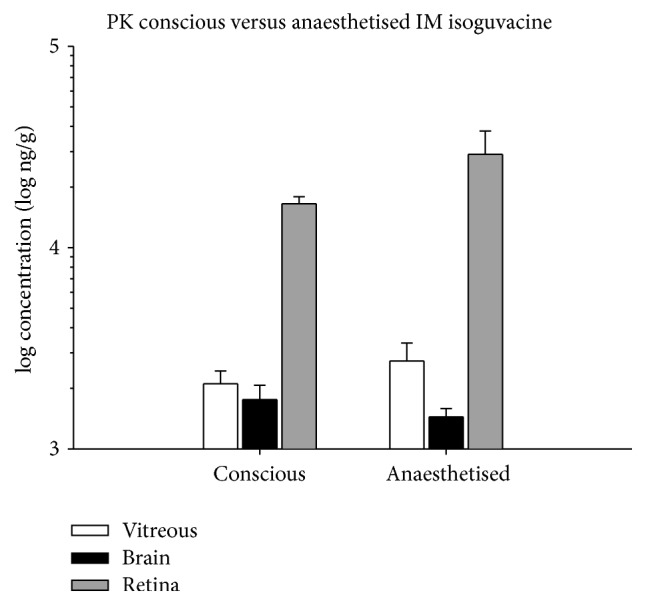
Pharmacokinetic analysis for IM injection of isoguvacine in conscious versus anaesthetised rats. Average ± SEM concentration (log ng/g) of isoguvacine in the vitreous (unfilled), brain (black), and retina (grey) 1.5 hours following IM administration of 30 mg/kg isoguvacine in conscious versus anaesthetised (ket : xyl) rats (*n* = 5 for each group).

Figure 5 compares isoguvacine concentration in the vitreous (white bars), brain (black bars), and retina (grey bars) following IM injection of isoguvacine in conscious and anaesthetised (ketamine : xylazine) rats. There was no significant difference in isoguvacine concentration between tissues collected following drug dosing in conscious or anaesthetised animals (vitreous: conscious 2107 ± 332, ket : xyl 2736 ± 619, *p* = 0.40, brain: conscious 1760 ± 310, ket : xyl 1442 ± 143, *p* = 0.38; retina: conscious 16525 ± 1386, ket : xyl 29080 ± 8870, *p* = 0.26, all ng/g).

PK analysis was also undertaken for groups of rats dosed with different concentrations of muscimol. However, as the concentrations of muscimol injected were much less than isoguvacine (see Section 2.6), the results were not significantly above the spectrometer's noise level (data not shown).

## 4. Discussion

### 4.1. Visual Electrophysiology in Conscious Rats for CNS Drug Penetrance Testing

We show proof-of-principle evidence indicating that light evoked ERG and/or VEP responses from conscious rats can be used to detect CNS drug penetrance. Consistent with previous findings that isoguvacine does not readily cross the blood neural barriers [35], IM injection of isoguvacine did not affect the ERG nor VEP in awake rats. When we deliberately bypassed the blood neural barriers (IV or ICV injections) isoguvacine produced significant ERG (Figure 1(b)) and VEP (Figure 3(b)) changes. On the other hand, muscimol produced ERG (Figures 1(c) and 1(d)) and VEP (Figures 3(c) and 3(d)) changes regardless of the route of injection, consistent with its greater capacity for CNS penetrance [35].  Table 1 summarises all the electrophysiology findings in this study. One factor to consider in intravitreal administration of the compounds is the possible effect of increased intraocular pressure (IOP) on retinal function. However, it has been shown that a temporary spike in IOP results in transient global depression of the ERG waveform [57], which is not seen in our data (Table 1). Therefore the findings reported here are most likely due to drug-driven responses.

In conscious rats, the presence of GABAa agonists in the retina did not affect photoreceptor and rod bipolar amplitudes (Figures 1(e) and 1(f)) but consistently decreased amplitudes of cone-mediated responses (Figure 1(g)). Kapousta-Bruneau [58] reported that bicuculline, a GABAa antagonist, increased the rat ERG b-wave, which is consistent with the b-wave reduction that we see following administration of GABAa agonists (Figure 1(f)). The Kapousta-Bruneau study [58] measured a change in mixed (rod and cone) driven b-waves. Our data would suggest that the amplitude attenuation reflects loss of cone-mediated bipolar cell responses (Figure 1(g)).

This preferential reduction in cone bipolar cell output may be explained by differences in the extent to which GABAa and GABAc receptors modulate rod and cone bipolar cell currents. Euler and Wässle [59] puffed GABA onto bipolar cells in isolated rat retina and reported that approximately 70% of the GABA current in rod bipolar cells was mediated by GABAc receptors (30% GABAa) as opposed to 20% in cone bipolar cells. Given this finding, GABAa agonists would be expected to produce greater changes in cone-mediated ERG (Figure 1(g)). However from the literature, it is not completely clear why rod bipolar sensitivity changes arise following administration of muscimol administration but not isoguvacine (Figure 1(h)). The literature reports that muscimol has high affinity for GABAa as well as GABAc receptors, whereas isoguvacine acts primarily as a GABAa receptor agonist [35, 36]. If muscimol modulates both GABAa and GABAc receptors then this would have a bigger effect on the modulatory effect of inhibition in rod bipolar cell currents where GABAc makes a proportionately larger contribution. Thus isoguvacine with its weaker agonism of GABAc receptors would be less likely to influence rod bipolar cell sensitivity.

Local isoguvacine and local/systemic muscimol administration resulted in timing delays in the VEP. In contrast systemic isoguvacine produced no significant changes to the waveform. These findings are consistent with the notion that isoguvacine has poor CNS penetrance compared with muscimol. It is worth noting that the rod pathway dominates the ERG response, whereas the cone pathway largely drives the VEPs returned by the current protocol. Nevertheless, the fact that the ERG and VEP show similar patterns of changes to these drug changes suggests that retinal electrophysiology in conscious rats may be a useful way to test for cortical drug penetrance.

In terms of the mechanisms for the slower VEPs, isoguvacine [60–62] and muscimol [63, 64] have been shown to decrease neuronal-firing rates by modulating N-methyl-D-aspartate pathways, which can induce a slowing of the waveform. However, GABAergic inhibition in the CNS also has complex interactions with other neurotransmitter systems such as acetylcholine, norepinephrine, and serotonin [65], all of which can affect the VEP [66]. We also saw an increase in VEP amplitude following muscimol administration. This may arise from altered inhibitory modulation that will result in an increase in brain activity consistent with Lancel et al. [67] who observed larger electroencephalograms in rats following systemic muscimol administration.

### 4.2. Ketamine : Xylazine Anaesthesia Confounds the Interpretation of Central Penetrance

Figures 2(a) and 2(c) show that IM injection of isoguvacine and muscimol in anaesthetised rats produced ERG waveforms quite different from those recorded following IM dosing in conscious rats. First, there was a reduction in photoreceptor amplitude with both isoguvacine and muscimol IM injections in anaesthetised rats, whereas in conscious rats there was no change. The pharmacokinetic data (Figure 5) shows that tissue concentration of isoguvacine was similar in conscious and anaesthetised rats, which argues against any anaesthesia-mediated increase in the permeability of blood neural barriers for isoguvacine. Furthermore, immunohistochemistry studies [37, 38] have localised GABAa receptors to cholinergic amacrine cells, dopaminergic amacrine cells, and bipolar cells but not photoreceptors. Thus the reduction in the a-wave is unlikely to be a direct effect of isoguvacine or muscimol on photoreceptors. Consistent with this hypothesis, we find that intravitreal injection of isoguvacine or muscimol (Figures 2(b) and 2(d)) did not produce a greater a-wave reduction. An alternative explanation is that systemic changes arising from IM administration of muscimol or isoguvacine in anaesthetised rats may contribute to a smaller photoreceptor signal. A number of systemic changes that can accompany general anaesthesia include a reduction in blood pressure [68] and temperature [24] which could confound expression of drug effects. It may be possible that the combination of ketamine, xylazine, and the inhibitory effect of GABAa agonists could depress general systemic function to a point that compromises photoreceptor function.

In order to consider postphotoreceptoral drug effects, ERG responses were normalised to a-wave output for each animal (see Supplementary Material, Figure S1) (see Supplementary Material available online at http://dx.doi.org/10.1155/2016/5801826). This approach shows that, for IM injection of isoguvacine in anaesthetised rats, the reduction in a-wave output can account for the subsequent b-wave attenuation. However, decreased a-wave output does not account for the paradoxical increase in rod-b-wave amplitude seen following IV injection of isoguvacine (larger, faster b-wave, Figure 2(b)). This is in stark contrast to the effect seen in conscious rats, where IV isoguvacine produced more subtle ERG changes. This unusual outcome is difficult to explain. Given that activation of GABAa and GABAc receptors leads to opposite effects on the ERG b-wave amplitude [69, 70] one could speculate that the presence of ketamine : xylazine modifies the effect of GABAa agonists employed here to be more GABAc active. Moreover, the different affinity of muscimol and isoguvacine for GABAa and GABAc receptors [35, 36] has the potential to change the balance of GABAa and GABAc mediated modulation of bipolar cell currents under anaesthetised conditions.

Similar to the ERG changes, isoguvacine and muscimol when injected into anesthetised rats produced VEPs changes different from those seen in conscious rats (Figure 4). In particular, in anaesthetised rats, direct delivery of isoguvacine or muscimol produced smaller waveforms, an effect not seen in conscious rats. Further work is needed to understand the mechanism by which ketamine and xylazine modify the effect of GABA agonists on both the ERG and VEP.

## 5. Conclusions

Recording of visually evoked responses from conscious rats can help us determine whether a drug has the ability to cross from the blood stream into the CNS. We provide proof-of-principle data in support for this idea using GABA agonists. The presence of ketamine : xylazine anaesthesia changes electrophysiology findings such that altered ERG waveforms are seen even with IM injection of a drug that is not centrally penetrant. Thus anaesthesia can lead to erroneous conclusions regarding a drugs central penetrance. These findings suggest that visual electrophysiology in conscious rats can be a useful method for CNS drug testing. The fact that the pattern of functional changes seen in the eye parallels those in the brain supports the notion that the retina may be a useful CNS biomarker.

Increased efficiency in preclinical drug testing is desperately needed for the drug industry to cope with the demands associated with longer life expectancy, and viable neurological biomarkers are a key factor to address this challenge. The retina, being a more accessible organ than the brain, may provide new avenues to service these needs.

## Supplementary Material

By normalising drug effects to the a-wave it is evident that with IM injection of isoguvacine in anaesthetized rats, the reduction in a-wave output can account for the subsequent b-wave attenuation. However, decreased a-wave output does not account for the paradoxical increase in rod b-wave amplitude seen following IV injection of isoguvacine.

## Figures and Tables

**Table 1:** Summary of GABAa agonist effects on the ERG and VEP in conscious and anaesthetised rats.

	IM isoguvacine		IV isoguvacine		ICV isoguvacine		IM muscimol		IV muscimol		ICV muscimol	
	Conscious	ket : xyl	Conscious	ket : xyl	Conscious	ket : xyl	Conscious	ket : xyl	Conscious	ket : xyl	Conscious	ket : xyl
(A) ERG parameters												
Photoreceptor amplitude	—	↓	—	—			—	↓	—	—		
Rod bipolar cell amplitude	—	↓	—	↑			—	↓	—	↓		
Cone amplitude	—	↓	↓	↓			↓	↓	↓	↓		
Photoreceptor sensitivity	—	—	—	—			—	↓	—	—		
Rod bipolar cell sensitivity	—	—	—	↑			↑	↑	↑	↑		
Cone bipolar implicit time	—	—	—	↑			—	↑	—	↑		

(B) VEP parameters												
P1-N1 amplitude	—	—			—	↓	↑	↑			↑	↓
P2-N1 amplitude	—	—			—	↓	↑	—			↑	—
N1 time	—	—			—	↑	↑	↑			—	↑
P2 time	—	—			↑	—	—	—			—	—

Arrows indicate the direction of effect compared to relevant baseline (conscious or anaesthetised); dashes indicate no significant effect. IM: intramuscular; IV: intravitreal; ICV: intracerebroventricular.

## References

[1] Pangalos M. N., Schechter L. E., Hurko O. (2007). Drug development for CNS disorders: strategies for balancing risk and reducing attrition. *Nature Reviews Drug Discovery*.

[2] Choi D. W., Armitage R., Brady L. S. (2014). Medicines for the mind: Policy-based “Pull” incentives for creating breakthrough CNS drugs. *Neuron*.

[3] Lutz W., Sanderson W., Scherbov S. (2008). The coming acceleration of global population ageing. *Nature*.

[4] Barnard N. D., Bush A. I., Ceccarelli A. (2014). Dietary and lifestyle guidelines for the prevention of Alzheimer's disease. *Neurobiology of Aging*.

[5] Sherratt M. J. (2013). Age-related tissue stiffening: cause and effect. *Advances in Wound Care*.

[6] Kola I., Landis J. (2004). Can the pharmaceutical industry reduce attrition rates?. *Nature Reviews Drug Discovery*.

[7] Sinn R., Wittbrodt J. (2013). An eye on eye development. *Mechanisms of Development*.

[8] Alm A., Tornquist P. (1985). Lactate transport through the blood-retinal and the blood-brain barrier in rats. *Ophthalmic Research*.

[9] Steuer H., Jaworski A., Elger B. (2005). Functional characterization and comparison of the outer blood-retina barrier and the blood-brain barrier. *Investigative Ophthalmology and Visual Science*.

[10] Törnquist P., Alm A. (1986). Carrier-mediated transport of amino acids through the blood-retinal and the blood-brain barriers. *Graefe's Archive for Clinical and Experimental Ophthalmology*.

[11] Baker M. L., Hand P. J., Wang J. J., Wong T. Y. (2008). Retinal signs and stroke: revisiting the link between the eye and brain. *Stroke*.

[12] Patton N., Aslam T., MacGillivray T., Pattie A., Deary I. J., Dhillon B. (2005). Retinal vascular image analysis as a potential screening tool for cerebrovascular disease: a rationale based on homology between cerebral and retinal microvasculatures. *Journal of Anatomy*.

[13] Qiu C., Cotch M. F., Sigurdsson S. (2008). Retinal and cerebral microvascular signs and diabetes: the age, gene/environment susceptibility-Reykjavik study. *Diabetes*.

[14] Wong T. Y., Klein R., Richey Sharrett A. (2002). Cerebral white matter lesions, retinopathy, and incident clinical stroke. *The Journal of the American Medical Association*.

[15] Gadea A., Lpez-Colom A. M. (2001). Glial transporters for glutamate, glycine, and GABA III. Glycine transporters. *Journal of Neuroscience Research*.

[16] Gadea A., López-Colomé A. M. (2001). Glial transporters for glutamate, glycine, and GABA: II. GABA transporters. *Journal of Neuroscience Research*.

[17] Gadea A., López-Colomé A. M. (2001). Glial transporters for glutamate, glycine and GABA I. Glutamate transporters. *Journal of Neuroscience Research*.

[18] MacCormick I. J., Czanner G., Faragher B. (2015). Developing retinal biomarkers of neurological disease: an analytical perspective. *Biomarkers in Medicine*.

[19] Frost S., Kanagasingam Y., Sohrabi H. (2013). Retinal vascular biomarkers for early detection and monitoring of Alzheimer's disease. *Translational Psychiatry*.

[20] Hosford D. A., Wang Y. (1997). Utility of the lethargic (lh/lh) mouse model of absence seizures in predicting the effects of lamotrigine, vigabatrin, tiagabine, gabapentin, and topiramate against human absence seizures. *Epilepsia*.

[21] Kupferberg H. (2001). Animal models used in the screening of antiepileptic drugs. *Epilepsia*.

[22] Lowson S., Gent J. P., Goodchild C. S. (1990). Anticonvulsant properties of propofol and thiopentone: comparison using two tests in laboratory mice. *British Journal of Anaesthesia*.

[23] Frishman L. J. (2006). *Origins of the Electroretinogram*.

[24] Weymouth A. E., Vingrys A. J. (2008). Rodent electroretinography: methods for extraction and interpretation of rod and cone responses. *Progress in Retinal and Eye Research*.

[25] Lavoie J., Illiano P., Sotnikova T. D., Gainetdinov R. R., Beaulieu J.-M., Hébert M. (2014). The electroretinogram as a biomarker of central dopamine and serotonin: potential relevance to psychiatric disorders. *Biological Psychiatry*.

[26] Eccles J. C. (1951). Interpretation of action potentials evoked in the cerebral cortex. *Electroencephalography and Clinical Neurophysiology*.

[27] Fahle M., Bach M., Heckenlively J. R., Arden G. B. (2006). Origin of the visual evoked potentials. *Principles and Practice of Clinical Electrophysiology of Vision*.

[28] Creel D., Dustman R. E., Beck E. C. (1974). Intensity of flash illumination and the visually evoked potential of rats, guinea pigs and cats. *Vision Research*.

[29] Galambos R., Szabó-Salfay O., Szatmári E., Szilágyi N., Juhász G. (2001). Sleep modifies retinal ganglion cell responses in the normal rat. *Proceedings of the National Academy of Sciences of the United States of America*.

[30] Meeren H. K. M., Van Luijtelaar E. L. J. M., Coenen A. M. L. (1998). Cortical and thalamic visual evoked potentials during sleep-wake states and spike-wave discharges in the rat. *Electroencephalography and Clinical Neurophysiology*.

[31] Nair G., Kim M., Nagaoka T. (2011). Effects of common anesthetics on eye movement and electroretinogram. *Documenta Ophthalmologica*.

[32] Charng J., Nguyen C. T., He Z. (2013). Conscious wireless electroretinogram and visual evoked potentials in rats. *PLoS ONE*.

[33] Jehle T., Ehlken D., Wingert K., Feuerstein T. J., Bach M., Lagrèze W. A. (2009). Influence of narcotics on luminance and frequency modulated visual evoked potentials in rats. *Documenta Ophthalmologica*.

[34] Guarino I., Loizzo S., Lopez L., Fadda A., Loizzo A. (2004). A chronic implant to record electroretinogram, visual evoked potentials and oscillatory potentials in awake, freely moving rats for pharmacological studies. *Neural Plasticity*.

[35] Krogsgaard-Larsen P. (1981). *γ*-Aminobutyric acid agonists, antagonists, and uptake inhibitors. Design and therapeutic aspects. *Journal of Medicinal Chemistry*.

[36] Johnston G. A., Chebib M., Hanrahan J. R., Mewett K. N. (2003). GABA(C) receptors as drug targets. *Current Drug Targets. CNS and Neurological Disorders*.

[37] Greferath U., Grunert U., Fritschy J. M., Stephenson A., Mohler H., Wassle H. (1995). GABAA receptor subunits have differential distributions in the rat retina: in situ hybridization and immunohistochemistry. *Journal of Comparative Neurology*.

[38] Sassoe-Pognetto M., Kirsch J., Grünert U. (1995). Colocalization of gephyrin and GABAA-receptor subunits in the rat retina. *Journal of Comparative Neurology*.

[39] Bosman L. W. J., Rosahl T. W., Brussard A. B. (2002). Neonatal development of the rat visual cortex: Synaptic function of GABAA receptor *α* subunits. *Journal of Physiology*.

[40] Warner D. O. (2000). Preventing postoperative pulmonary complications: the role of the anesthesiologist. *Anesthesiology*.

[41] Cowey A., Franzini C. (1979). The retinal origin of uncrossed optic nerve fibres in rats and their role in visual discrimination. *Experimental Brain Research*.

[42] Tsai T. I., Bui B. V., Vingrys A. J. (2014). Effect of acute intraocular pressure challenge on rat retinal and cortical function. *Investigative Ophthalmology and Visual Science*.

[43] He Z., Bui B. V., Vingrys A. J. (2006). The rate of functional recovery from acute IOP elevation. *Investigative Ophthalmology and Visual Science*.

[44] Nixon P. J., Bui B. V., Armitage J. A., Vingrys A. J. (2001). The contribution of cone responses to rat electroretinograms. *Clinical and Experimental Ophthalmology*.

[45] Hughes A. (1979). A schematic eye for the rat. *Vision Research*.

[46] Pardridge W. M. (2011). Drug transport in brain via the cerebrospinal fluid. *Fluids and Barriers of the CNS*.

[47] Wong V. H. Y., Vingrys A. J., Bui B. V. (2011). Glial and neuronal dysfunction in streptozotocin-induced diabetic rats. *Journal of Ocular Biology, Diseases, and Informatics*.

[48] Haynes J. J., Jones H., Gibson D., Clark G. T. (2011). Bioanalytical determination of unstable endogenous small peptides: RFRP3 & its metabolites in rat blood. *Bioanalysis*.

[49] Sillén H., Cook M., Davis P. (2010). Determination of ticagrelor and two metabolites in plasma samples by liquid chromatography and mass spectrometry. *Journal of Chromatography B: Analytical Technologies in the Biomedical and Life Sciences*.

[50] Hood D. C., Birch D. G. (1994). Rod phototransduction in retinitis pigmentosa: estimation and interpretation of parameters derived from the rod a-wave. *Investigative Ophthalmology and Visual Science*.

[51] Lamb T. D., Pugh E. N. (1992). A quantitative account of the activation steps involved in phototransduction in amphibian photoreceptors. *Journal of Physiology*.

[52] Cideciyan A. V., Jacobson S. G. (1996). An alternative phototransduction model for human rod and cone ERG *α*-waves: normal parameters and variation with age. *Vision Research*.

[53] Hood D. C., Bircht D. G. (1990). The A-wave of the human electroretinogram and rod receptor function. *Investigative Ophthalmology and Visual Science*.

[54] Nguyen C. T. O., Vingrys A. J., Bui B. V. (2008). Dietary omega-3 fatty acids and ganglion cell function. *Investigative Ophthalmology and Visual Science*.

[55] Saszik S. M., Robson J. G., Frishman L. J. (2002). The scotopic threshold response of the dark-adapted electroretinogram of the mouse. *The Journal of Physiology*.

[56] van Hulzen Z. J. M., Coenen A. M. L. (1984). Photically evoked potentials in the visual cortex following paradoxical sleep deprivation in rats. *Physiology and Behavior*.

[57] Bui B. V., Edmunds B., Cioffi G. A., Fortune B. (2005). The gradient of retinal functional changes during acute intraocular pressure elevation. *Investigative Ophthalmology and Visual Science*.

[58] Kapousta-Bruneau N. V. (2000). Opposite effects of GABA(A) and GABA(C) receptor antagonists on the b- wave of ERG recorded from the isolated rat retina. *Vision Research*.

[59] Euler T., Wässle H. (1998). Different contributions of GABAA and GABAC receptors to rod and cone bipolar cells in a rat retinal slice preparation. *Journal of Neurophysiology*.

[60] Kitai S. T., Shepard P. D., Callaway J. C., Scroggs R. (1999). Afferent modulation of dopamine neuron firing patterns. *Current Opinion in Neurobiology*.

[61] Lobb C. J., Wilson C. J., Paladini C. A. (2010). A dynamic role for GABA receptors on the firing pattern of midbrain dopaminergic neurons. *Journal of Neurophysiology*.

[62] Paladini C. A., Iribe Y., Tepper J. M. (1999). GABA(A) receptor stimulation blocks NMDA-induced bursting of dopaminergic neurons in vitro by decreasing input resistance. *Brain Research*.

[63] Celada P., Paladini C. A., Tepper J. M. (1999). GABAergic control of rat substantia nigra dopaminergic neurons: role of globus pallidus and substantia nigra pars reticulata. *Neuroscience*.

[64] Antkowiak B. (1999). Different actions of general anesthetics on the firing patterns of neocortical neurons mediated by the GABA(A) receptor. *Anesthesiology*.

[65] Matsumoto R. R. (1989). GABA receptors: are cellular differences reflected in function?. *Brain Research Reviews*.

[66] Hetzler B. E., Oaklay K. E. (1981). Dose effects of pentobarbital on evoked potentials in visual cortex and superior colliculus of the albino rat. *Neuropharmacology*.

[67] Lancel M., Cronlein T. A., Faulhaber J. (1996). Role of GABAA receptors in sleep regulation. Differential effects of muscimol and midazolam on sleep in rats. *Neuropsychopharmacology*.

[68] Sanford T. D., Colby E. D. (1980). Effect of xylazine and ketamine on blood pressure, heart rate and respiratory rate in rabbits. *Laboratory Animal Science*.

[69] Dong C.-J., Hare W. A. (2002). GABAc feedback pathway modulates the amplitude and kinetics of ERG b-wave in a mammalian retina in vivo. *Vision Research*.

[70] Gottlob I., Wündsch L., Tuppy F. K. (1988). The rabbit electroretinogram: effect of GABA and its antagonists. *Vision Research*.

