# Supplementary material for: Retinal Electrophysiology Is a Viable Preclinical Biomarker for Drug Penetrance into the Central Nervous System
Source: J Ophthalmol. 2016 Apr 27;2016:5801826. doi: 10.1155/2016/5801826 (PMC4863103; doi:10.1155/2016/5801826)
Supplement: Supplementary file 1 — By normalising drug effects to the a-wave it is evident that with IM injection of isoguvacine in anaesthetized rats, the reduction in a-wave output can account for the subsequent b-wave attenuation. However, decreased a-wave output does not account for the paradoxical increase in rod b-wave amplitude seen following IV injection of isoguvacine. [file 5801826.f1.pdf]

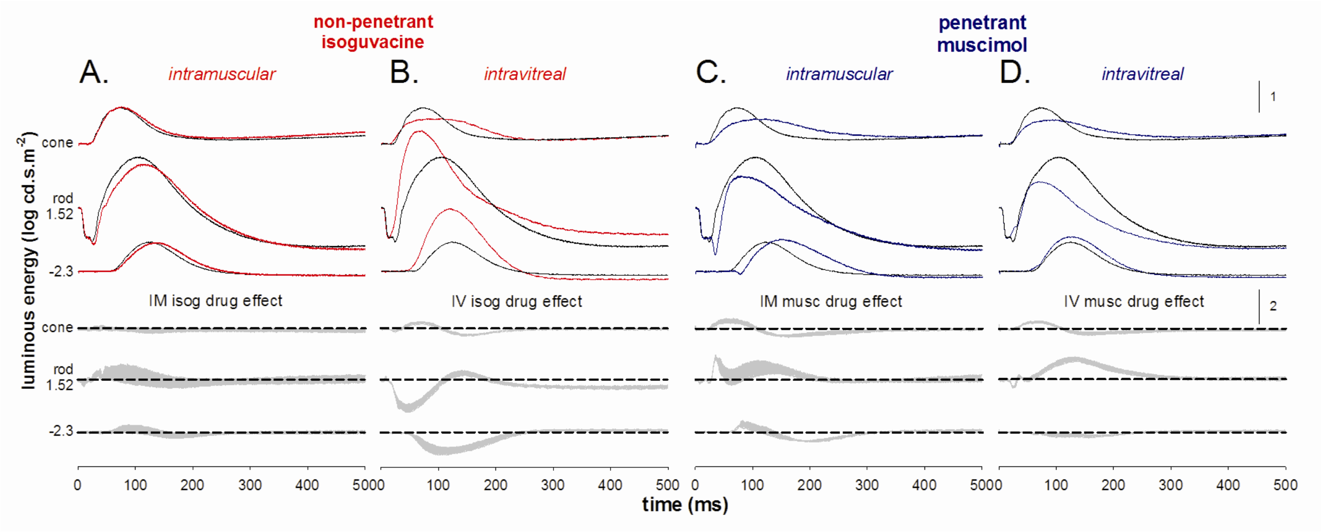

Figure S1: Effect of isoguvacine and muscimol on post-receptoral components of the retinal waveforms recorded from anaesthetised rats. Response across luminous energies are normalised by the a-wave (n = 5 for each drug and injection route). **A.** Average baseline (black) and post-IM isoguvacine (red) rod and cone ERG waveforms. The raw data are shown in Figure 2. The lower panels show the average residuals (± 95% CI in grey) for the difference of IM isoguvacine to baseline (i.e. drug effect), dashed lines indicate a residual of zero indicating no effect beyond that found for the a-wave. **B.** Normalised IV isoguvacine and baseline, details as per Panel A. **C.** Normalised IM muscimol and baseline, details as per Panel A. **D.** Normalised IV muscimol and baseline, details as per Panel A.
